# Editorial: The Ischemic Penumbra: Still the Target for Stroke Therapies?

**DOI:** 10.3389/fneur.2015.00085

**Published:** 2015-04-22

**Authors:** Argye E. Hillis, Jean-Claude Baron

**Affiliations:** ^1^Department of Neurology, Johns Hopkins University School of Medicine, Baltimore, MD, USA; ^2^Department of Physical Medicine and Rehabilitation, Johns Hopkins University School of Medicine, Baltimore, MD, USA; ^3^Department of Cognitive Science, Johns Hopkins University, Baltimore, MD, USA; ^4^Clinical Neurosciences, University of Cambridge, Cambridge, UK; ^5^INSERM U894, Centre de Psychiatrie et Neurosciences, Hôpital Sainte-Anne, Sorbonne Paris Cité, Paris, France

**Keywords:** acute ischemic stroke, penumbra, MRI perfusion, CT perfusion, tissue plasminogen activator

The ischemic penumbra refers to tissue at risk of infarction where perfusion is inadequate to support neuronal function, but just adequate to maintain cell viability (1). This dysfunctional, but salvageable tissue has been the target of all acute stroke therapies (2), and this concept underpinned the successful trials of intravenous thrombolysis using t-PA (3). Advanced imaging, including diffusion-weighted imaging (DWI) and perfusion-weighted imaging (PWI) MR and CT perfusion (CTp), was developed to rapidly identify stroke patients with still present penumbra, who were thought to be the best candidates for reperfusion therapies. However, early studies, using different methods for identifying penumbra, different measures of outcome, and different time-windows have not consistently confirmed the benefit of selecting treatment candidates on the basis of imaged penumbra. Therefore, some outstanding questions surround the optimal modality for imaging the penumbra, the most reliable thresholds in each modality, how long the penumbra can be maintained under what subject-specific circumstances, and the functional significance of persistent penumbra. These questions have taken on particular importance in light of the results of five recently completed randomized clinical trials showing benefit of endovascular treatment of stroke, when patients are carefully selected and treated on a timely basis. These trials include MR CLEAN (4), ESCAPE (5); EXTEND-IA (6), and two trials that have not been published, but the results of which have been presented at the International Stroke Conference [SWIFT PRIME (7) and REVASCAT^1^]. These trials have used different criteria to select patients for treatment, including different modalities of imaging (CT vs. MRI), but those that have shown the highest odds of favorable functional outcome have selected patients on the basis of having both a small core infarct, and either large volume of penumbral tissue (“tissue at risk”) (6, 7) or the presence of moderate–good collateral circulation (5) that would support penumbral tissue in the face of proximal occlusion.

These recent studies, together with an earlier successful pilot trial of another thrombolytic agent that used MR-based selection of target penumbral patients (8) have shown the importance of selecting patients on the basis of the presence of penumbral tissue, but underscore the urgency of defining appropriate thresholds with imaging that can be obtained swiftly in order to maximize the efficiency of intervention. While the gold standard for both irreversibly ischemic core and penumbra has been defined by PET (2), PET cannot be obtained rapid enough to provide a practical guide for acute stroke treatment. Some centers are able to obtain rapid MRI, while most will rely likely on multiphase CT angiogram and/or CTp to guide intervention. It is critical that the stroke field adopts valid and reliable thresholds using any of these modalities to select candidates for intervention. Toward this goal, two MR vs. PET back-to-back studies have proposed validated MR-perfusion thresholds, based on small samples (9, 10). This Research Topic consists of a set of papers that addresses some of the controversies and intriguing questions that remain.

Kaesemann and colleagues (11) evaluated the impact of severe extracranial ICA stenosis on MRI measures of penumbra in patients with middle cerebral artery occlusion who were imaged within 4.5 h of onset. They evaluated core infarct volumes, mean transit time (MTT), *T*_max_, cerebral blood volume (CBV), and cerebral blood flow (CBF) maps, as well as tissue at risk (*T*_max_ >6 – infarct volume). The presence of the additional extracranial stenosis did not affect measured infarct volume, MTT, *T*_max_, or tissue at risk, but had a small influence on CBV. They hypothesized that extracranial stenosis may lead to ischemic preconditioning that results in improved collateral circulation and a consequent increase in CBV in the presence of acute stroke.

Wouters and co-workers (12) discuss proposed imaging criteria, including diffusion-FLAIR mismatch, for selecting patients who wake up with stroke and or have unknown onset. They point out that there are currently no data for selecting one set of criteria over another, but argue that identifying patients who have penumbral tissue with imaging should allow intravenous and/or endovascular treatment of many of these patients.

Leigh and colleagues (13) hypothesized that the conflicting conclusions from two large endovascular trials, MR RESCUE and DEFUSE 2, regarding the usefulness of MRI diffusion and perfusion imaging for selecting candidates for treatment were due to differences in definitions of core infarct and “tissue at risk.” MRI scans from patients evaluated for endovascular therapy were processed using the methods published in the two trials. The volume of core infarct was consistently smaller when defined by MR RESCUE criteria than DEFUSE 2 criteria. The volume of tissue at risk was consistently larger when defined by the MR RESCUE criteria than DEFUSE 2 criteria. When these volumes were used to classify MRI scans, 9 out of 12 patients (75%) were classified as having salvageable tissue by MR RESCUE, while only 4 out of 12 patients (33%) were classified as having salvageable tissue by DEFUSE 2 criteria.

Marsh and co-workers (14) present two patients who underwent endovascular treatment with very different outcomes. They argue that robust collateral circulation supported a prolonged penumbra in the patient who showed minimal progression to infarct and outstanding functional outcome despite a delay in treatment.

Agarwal and colleagues (15) compared quantitative hemodynamic measures of CTp (volumes of penumbra defined by CBF, or PenCBF, and penumbra defined by MTT, or PenMTT), a visually defined CBF/CBV ASPECTS ratio, and a visually rated collateral circulation on CTA. They found that both PenCBF and PenMTT showed trends to decrease with increased time since onset. The CBF/CBV ASPECTS ratio, which was related to the PenCBF, significantly decreased with increased time since onset. In contrast, the rating of collateral response was not related to time since onset. These results raise some questions as to whether the presence of collaterals can be used as a surrogate for the presence of penumbral tissue in selecting candidates for intervention.

Campbell and colleagues (16) discuss challenges of imaging the penumbra and provide useful guidelines. They also discuss scenarios in which recanalization and reperfusion are discordant: both cases in which there is recanalization without reperfusion and reperfusion without recanalization (via enhanced retrograde collateral flow). Finally, they discuss infarct growth and the fact that there is sometimes persistent hypoperfusion that accounts for clinical deficits.

Motta et al. (17) investigated the clinical consequences of persistent hypoperfusion. They found that uninfarcted but hypoperfused tissue, with a threshold of 4–5.9 s delay on time-to-peak (TTP) maps on PWI occasionally persists for days and is associated with cognitive deficits such as aphasia or neglect. Furthermore, change in volume of hypoperfused tissue of 4–5.9 s delay and change in volume of ischemic tissue on DWI over the first few days were independently associated with change in cognitive function. Sebastian et al. (18) also show that persistent cortical hypoperfusion caused by arterial stenosis can cause aphasia or neglect (in cases of purely thalamic infarct), although some cases of aphasia after thalamic stroke are likely due to cortical dysfunction (diaschisis) in the absence of hypoperfusion caused by arterial stenosis.

Finally, Scalzo and colleagues (19) argue that there are likely to be detailed features of CT and MRI that are not currently tapped, which may provide useful information for guiding stroke intervention. Use of computer vision and machine learning to incorporate aspects of imaging data that we may not realize are relevant may yield data-driven approaches to clinical decision-support.

This Research Topic thus addresses important and timely concerns surrounding the issue of how the ischemic penumbra can best be rapidly identified on imaging in order to contribute to management of acute stroke.

## Conflict of Interest Statement

The authors declare that the research was conducted in the absence of any commercial or financial relationships that could be construed as a potential conflict of interest.

## Footnotes

^1^Davalos A, Jovin T. REVASCAT – clinicalTrials.gov, NCT01692379
